# Fit-Seq2.0: An Improved Software for High-Throughput Fitness Measurements Using Pooled Competition Assays

**DOI:** 10.1007/s00239-023-10098-0

**Published:** 2023-03-06

**Authors:** Fangfei Li, Jason Tarkington, Gavin Sherlock

**Affiliations:** Department of Genetics, Stanford University, Stanford, USA

**Keywords:** Barcode, Fitness, Pooled growth, High-throughput phenotyping

## Abstract

The fitness of a genotype is defined as its lifetime reproductive success, with fitness itself being a composite trait likely dependent on many underlying phenotypes. Measuring fitness is important for understanding how alteration of different cellular components affects a cell’s ability to reproduce. Here, we describe an improved approach, implemented in Python, for estimating fitness in high throughput via pooled competition assays.

## Introduction

The fitness of an organism is dependent on many traits which act in concert to determine its reproductive success. Often a single trait can have an outsized role in determining fitness and in these cases, it may be appropriate to use these traits as easily quantifiable proxies of fitness. However, such approaches are limited in that they only measure a single component of fitness and in many cases, other unmeasured components of fitness may be relevant. A better approach involves directly competing genotypes against one another and then inferring fitness based on changes in genotype frequency. This approach captures all components of fitness simultaneously, allowing fitness instead of a proxy for fitness to be quantified. Previous approaches to competitive fitness assays have utilized differentially marked strains to perform pairwise fitness assays (Lenski et al. 1991); however, these approaches are limited in the throughput with which they can be performed. Some modest improvement to the throughput of competitive fitness assays has been achieved by utilizing fluorescently tagged lineages which allows the size of a lineage to be counted via the fluorescent signal instead of plating (Kao and Sherlock 2008; DeLuna et al. 2008).

Advances in molecular biology led to significant improvement to throughput by instead using DNA barcodes to mark and track lineages (Winzeler et al. 1999; Giaever et al. 2002) instead of fluorescent or other markers. At first, this involved transforming unique barcodes into known variants and then pooling 100 s of these barcoded variants into a library. These barcode tags could then be amplified via PCR and counted via hybridization to high-density arrays containing tag complements (Winzeler et al. 1999). A barcoded population could then be grown in a chemostat or via serial dilution for a finite number of generations and the fitness of each barcoded lineage could be inferred by tracking the changes in the barcode frequency over time. This approach was initially applied to yeast deletion libraries to test the fitness effects of 100 s of gene deletions across different environments (Winzeler et al. 1999; Giaever et al. 2002; Steinmetz et al. 2002). Later advances utilized high-throughput sequencing to count barcodes instead of hybridization arrays allowing for better quantification of barcode lineage frequencies within a population (Smith et al. 2009). Improved sequencing throughput allowed for the use of larger barcoded libraries, containing $\sim$500,000 barcodes, on an isogenic background to measure the fitness effects of *de novo* mutations that arise during the course of evolution (Levy et al. 2015).

Pooled competition assays using amplicon sequencing are becoming an increasingly common method for phenotyping large pools of variants simultaneously. This type of high-throughput phenotyping has applications in the characterization of *in vivo* adaptive mutations (Levy et al. 2015; Venkataram et al. 2016; Li et al. 2019), genetic interaction screening (Du et al. 2017; Jaffe et al. 2017; Díaz-Mejía et al. 2018), protein–protein interaction screening (Yachie et al. 2016; Celaj et al. 2017; Schlect et al. 2017), CRISPR screens (Koike-Yusa et al. 2014; Shalem et al. 2014; Smith et al. 2016; Zhu et al. 2021; Joung et al. 2022), deep mutational scanning (Fowler and Fields 2014), transposon mutagenesis screening (van Opijnen et al. 2009; Michel et al. 2017; Price et al. 2018), deletion collection screening (Smith et al. 2010; Li et al. 2011), rescue screening (Ho et al. 2009), protein cost measurements (Frumkin et al. 2017), and QTL mapping (Nguyen Ba et al. 2022; Matsui et al. 2022). A typical way of analyzing the data generated in these experiments is the fold enrichment, by utilizing two time points and estimating fitness from the change in barcode frequency between these two time points, such as MAGeCK (Li et al. 2014), despite known biases that are introduced when employing this type of method (Li et al. 2018). The fold enrichment method provides an accurate ranked fitness for each barcoded lineage; however, these fitness estimates are biased and cannot be compared across experiments because they are highly sensitive to the presence of the other genotypes in the pool and the duration of the experiment (Li et al. 2018). This problem is highlighted by the fact that two researchers could perform the exact same experiment differing only in the number of generations and many variants would be enriched in the shorter experiment that would be depleted in the longer one. This happens because as the mean fitness of the population increases, genotypes with fitness that were once greater than the mean fitness could now be lower than the mean fitness, resulting in their frequencies going from increasing to decreasing.

We have previously demonstrated that fitness estimates can be improved using a method we call Fit-Seq which uses multiple time points to optimize fitness estimates via a likelihood maximization method so that expected lineage trajectories match the observed data (Li et al. 2018). This method effectively eliminates the bias in fitness estimates that is introduced by fold enrichment-based methods. When using Fit-Seq to estimate fitness, the population mean fitness is taken into consideration, meaning that the estimated fitness of variants are approximately the same regardless of the duration of the competition experiment. Here, we describe several improvements we have made to this method (which we refer as Fit-Seq2.0) and show that Fit-Seq2.0 results in improved estimates of the fitness when it is used to analyze a simulated dataset.

There are four main improvements of Fit-Seq2.0 compared with Fit-Seq. First, a more accurate likelihood function is defined in Fit-Seq2.0, which models various sources of noise more precisely, and thus enable us to estimate the fitness more accurately. Second, a better optimization algorithm is employed in the maximization of the likelihood function. Third, in addition to estimating the fitness as in Fit-Seq, Fit-Seq2.0 also gives an estimated initial cell number for each lineage, which also enables a more accurate estimation for the lineage trajectory. Additionally, Fit-Seq2.0 is implemented in Python with an option of parallel computing, compared with Fit-Seq which was non-parallelized and implemented in MATLAB, making Fit-Seq2.0 more accessible to a broader audience and resulting in a shorter run time.

## Methods

### Algorithm

Before introducing the algorithm, we first define a list of notations. Let $t_0, t_1, \ldots , t_K$ be a list of the sequencing time points, $r_k$ be the read number of a lineage at time point $t_k$, and $n_k$ be the cell number at the bottleneck of a lineage at time point $t_k$. Let $R_k$ be the total read depth of all lineages at time point $t_k$, and $N_k$ be the total number of cells at the bottleneck at time point $t_k$. Let *s* be the fitness of a lineage. Here, we use Malthusian fitness, which is defined as the exponential growth rate of a lineage when grown independently. Let ${\bar{s}}(t)$ be the mean fitness of the population of all lineages at time *t*. In Fit-Seq, we used an iterative approach. Specifically, we first made an initial estimation of the mean fitness ${\bar{s}}(t_k)$ at each sequencing time points $t_k$ by log-linear regression using the read number of the first two time points $r_0$ and $r_1$. Then for an observed lineage trajectory data $\{r_k\}$, we defined the likelihood function as the joint probability distribution of the read number $\{r_k\}$ given the fitness *s*,

1 $$\begin{aligned}{} & {} p(r_0,\ldots ,r_K \mid s) = p(r_0 \mid s) \, p(r_1 \mid r_0, s)\nonumber \\ {}{} & {} \quad \cdots p(r_K \mid r_{K-1}, s). \end{aligned}$$

The term $p(r_k \mid r_{k-1},s)$ for $1 \le k \le K$ on the right side of Equation (1) represents the theoretical distribution for the number of reads at the current time point $t_k$ conditioned on the previous time point $r_{k-1}$ and the fitness *s*. It is defined based on a birth-branching process (Levy et al. 2015),

2 $$\begin{aligned}&p(r_k \mid r_{k-1},s)= \sqrt{\frac{\left( r_{k-1} {\mathscr {E}}_k R_k /R_{k-1}\right) ^{1/2}}{4\pi \kappa r_k^{3/2}}} \nonumber \\ {}&\exp \left[ -\frac{\left( \sqrt{r}_k - \sqrt{r_{k-1} {\mathscr {E}}_kR_k /R_{k-1}}\right) ^2}{\kappa }\right].\end{aligned}$$

Here, $\kappa$ is a noise parameter capturing half of per-read variance in offspring number from time point $t_{k-1}$ to $t_k$, which accounts for the noise introduced by cell growth, cell transfer, genomic DNA extraction, PCR, and sequencing (Levy et al. 2015). ${\mathscr{E}}_k$ is a term that accounts for the change in frequency of a lineage due to the mean fitness and the fitness of the lineage between two successive time points, which is defined as

3 $$\begin{aligned} {\mathscr {E}}_k = \exp \left[ \left( t_k - t_{k-1} \right) s - \int _{t_{k-1}}^{t_k} {\bar{s}}(\eta )d\eta \right] . \end{aligned}$$

Since we only infer the mean fitness at time points that are sequenced, we linearly interpolate ${\bar{s}}(t)$ between two successive sequenced time points. We then found the value of *s* that maximizes the likelihood function $p(r_0, \ldots , r_K \mid s)$ and used the optimal value of *s* as the estimate for the fitness to update the mean fitness ${\bar{s}}(t_k)$ at each time point $t_k$ by

4 $$\begin{aligned} {\bar{s}}(t_k) = \sum \limits _i s_i f_{i, t_k}, \end{aligned}$$

with $s_i$ being the optimal fitness of lineage *i*, and $f_{i, t_k}$ being the read frequency of lineage *i* at time point $t_k$. We repeated the optimization process, until the sum of the optimal likelihood value of all lineages does not increase.

However, it should be emphasized that the likelihood function in Fit-Seq is approximated by Equation (1), which is less accurate. In fact, the distribution of the read number $r_k$ directly depends on the cell number $n_k$, rather than on $r_{k-1}$. To be more strict, we should instead factorize the joint probability distribution of the cell number $\{n_k\}$ as,

5 $$\begin{aligned}{} & {} p(n_1,\ldots ,n_K \mid n_0, s) \nonumber \\ {}{} & {} = p(n_1 \mid n_0, s) \, p(n_2 \mid n_1, s) \cdots p(n_K \mid n_{K-1}, s). \end{aligned}$$

In Fit-Seq2.0, we use the same iterative strategy as in Fit-Seq. However, we set the initial mean fitness to zero and redefine the likelihood function as the joint probability distribution of the read number $\{r_k\}$ given the initial cell number $n_0$ and the fitness *s*,

6 $$\begin{aligned}{} & {} p(r_0,\ldots ,r_K \mid n_0, s) \nonumber \\ {}{} & {}= p(r_0 \mid n_0) \int \prod _{k=1}^K p(n_k \mid n_{k-1},s) \, p(r_k \mid n_k) \,d n_1 \cdots d n_K, \end{aligned}$$

with

7 $$\begin{aligned}&p(n_k \mid n_{k-1},s) \nonumber \\ {}& \approx \sqrt{\frac{\left( n_{k-1} {\mathscr {E}}_k\right) ^{1/2}}{4\pi c_k n_k^{3/2}}} \exp \left[ -\frac{\left( \sqrt{n}_k - \sqrt{n_{k-1} {\mathscr {E}}_k}\right) ^2}{c_k}\right] , \nonumber \\ {}& 1 \le k \le K, \end{aligned}$$

8 $$\begin{aligned}&p(r_k \mid n_k) \nonumber \\ {}& \approx \sqrt{\frac{\left( n_k R_k/N_k\right) ^{1/2}}{4\pi \beta _k r_k^{3/2}}} \exp \left[ -\frac{\left( \sqrt{r}_k - \sqrt{n_k R_k/N_k}\right) ^2}{\beta _k}\right], \nonumber \\ {}& 0 \le k \le K. \end{aligned}$$

Here, $p(n_k \mid n_{k-1},s)$ represents the theoretical distribution for the number of cells at the current time point $t_k$ conditioned on the previous time point $n_{k-1}$ and the fitness *s*, which considers the noise introduced by cell growth and cell transfer. It is defined based on a birth-branching process with per-individual offspring number variance per growth cycle $2c_k = 2$. $p(r_k \mid n_k)$ represents the theoretical distribution for the number of reads at the current time point $t_k$ conditioned on the number of cells at the current time point, which considers the noise introduced by genomic DNA extraction, PCR, and sequencing. It can also be characterized as a branching process, with $2\beta _k$ being the per-read variance. In our simulated model, $2\beta$ can be calculated approximately as the sum of ${\bar{r}}_k / {\bar{n}}_k$ (reverse process of dilution, which approximately follows the negative binomial distribution), ${\bar{r}}_k / n_{\textrm{DNA}}$ (genomic DNA extraction), ${\bar{r}}_k / n_{\textrm{DNA}}$ (PCR), and 1 (sequencing). Here, ${\bar{r}}_k$ is the average read number per lineage at time point $t_k$ (${\bar{r}}_k \in \{20, 50, 100\}$ in simulation). ${\bar{n}}_k$ is the average cell number per lineage at the bottleneck at $t_k$ (${\bar{n}}_k = 100$ in simulation). $n_{\textrm{DNA}}$ is average genomic DNA copy number per lineage at $t_k$ ($n_{\textrm{DNA}}=500$ in simulation). Thus, in our simulations, $\beta \approx ({\bar{r}}_k / {\bar{n}}_k + 2{\bar{r}}_k / n_{\textrm{DNA}} + 1)/2$ takes the value that approximately ranges from 0.57 to 0.85.

Unlike Fit-Seq, where the likelihood function (Equation (1)) is defined conditionally on a single variable, i.e., the fitness *s*, the likelihood function in Fit-Seq2.0 (Equation (6)) is conditioned on both the fitness *s* and the initial cell number $n_0$. This enables us to estimate both the values of *s* and $n_0$ simultaneously in Fit-Seq2.0. In principle, evaluating the likelihood function in Fit-Seq2.0 involves a high dimensional integral over each of the *K* variables $n_1, n_2, \ldots , n_K$, which is impractical. Here, we take advantage of the form of $p(n_k \mid n_{k-1},s)$ and $p(r_k \mid n_k)$ (Equations (7) and (8)) to calculate the approximate likelihood function without high dimensional integration. Since our final goal is to find the optimal *s* and $n_0$ that maximize the likelihood function $p(r_0,\ldots ,r_K \mid n_0, s)$, we only keep the exponent that dominates the overall shape of the distribution in Equations (7) and (8), which yields,

9 $$\begin{aligned}&p(n_k \mid n_{k-1},s) \approx \exp \left[ -\frac{\left( \sqrt{n}_k - \sqrt{n_{k-1} {\mathscr {E}}_k}\right) ^2}{c_k}\right] , \nonumber \\ {}& 1 \le k \le K, \end{aligned}$$

10 $$\begin{aligned}&p(r_k \mid n_k) \approx \exp \left[ -\frac{\left( \sqrt{r}_k - \sqrt{n_k R_k/N_k}\right) ^2}{\beta _k}\right] , \nonumber \\ {}& 0 \le k \le K. \end{aligned}$$

Therefore, the likelihood function becomes

11 $$\begin{aligned}&p(r_0, \ldots , r_K \mid s, n_0) \nonumber \\&= p(r_0 \mid n_0) \nonumber \\ {}&\quad \int \exp \left[ -\sum _{k=1}^K \left( \frac{\left( \sqrt{n}_k - \sqrt{n_{k-1} {\mathscr {E}}_k}\right) ^2}{c_k} + \frac{\left( \sqrt{r}_k - \sqrt{n_k R_k/N_k}\right) ^2}{\beta _k} \right) \right] \nonumber \\ {}&\quad d n_1 \cdots d n_K. \end{aligned}$$

For the integral in Equation (11), we can use the maximum of the integrand to approximate its value instead of direct integration. Specifically, we define $\nu _k = \sqrt{n_k}$, $\gamma _k=\sqrt{r_k}$ , and $\rho _k = \sqrt{R_k/N_k}$. Then, we can find the values of $\nu _1,\ldots ,\nu _K$ that maximize the integrand, which becomes

12 $$\begin{aligned} \exp \left[ -\sum _{k=1}^K \left( \frac{\left( \nu _k - \sqrt{{\mathscr {E}}_k} \nu _{k-1}\right) ^2}{c_k} + \frac{\left( \rho _k \nu _k - \gamma _k \right) ^2}{\beta _k} \right) \right] . \end{aligned}$$

Since the exponent in the integrand is quadratic in $\nu _k$, we can maximize it by solving a set of *K* equations linear in the $\nu _k$,

13 $$\begin{aligned} \left\{ \begin{aligned}&\left( \frac{1}{c_k} + \frac{\rho _k^2}{\beta _k} + \frac{{\mathscr {E}}_{k+1}}{c_{k+1}} \right) \nu _k - \frac{\sqrt{{\mathscr {E}}_k}}{c_k} \nu _{k-1} - \frac{\sqrt{{\mathscr {E}}_{k+1}}}{c_{k+1}} \nu _{k+1} = \frac{\rho _k \gamma _k}{\beta _k}, \quad k = 1,\ldots , K-1, \\&\left( \frac{1}{c_K} + \frac{\rho _{K}^2}{\beta _K}\right) \nu _K - \frac{\sqrt{{\mathscr {E}}_K}}{c_K} \nu _{K-1} = \frac{\rho _K \gamma _K}{\beta _K}. \end{aligned} \right. \end{aligned}$$

This set of constraints can be written in matrix format below,

14 $$\begin{aligned} \left( \begin{matrix} m_{1,1} &{} m_{1,2} &{} \cdots &{} m_{1,K}\\ m_{2,1} &{} m_{2,2} &{} \cdots &{} m_{2,K}\\ \vdots &{} \vdots &{} \ddots &{} \vdots \\ m_{K,1} &{} m_{K,2} &{} \cdots &{} m_{K,K}\\ \end{matrix} \right) \left( \begin{matrix} \nu _1 \\ \nu _2 \\ \vdots \\ \nu _K \end{matrix} \right) = \left( \begin{matrix} b_1 \\ b_2 \\ \vdots \\ b_K \end{matrix} \right) , \end{aligned}$$

with

15 $$\begin{aligned} m_{i,j} = \left\{ \begin{aligned}&\frac{1}{c_i} + \frac{\rho _i^2}{\beta _i} + \frac{{\mathscr {E}}_{i+1}}{c_{i+1}}, \quad i = 1, \ldots , K-1, \quad j=i, \\&\frac{1}{c_i} + \frac{\rho _i^2}{\beta _i}, \quad i = K, \quad j = i, \\&-\frac{\sqrt{{\mathscr {E}}_{i+1}}}{c_{i+1}}, \quad i= 1, \ldots , K-1, \quad j=i+1 \\&-\frac{\sqrt{{\mathscr {E}}_i}}{c_i}, \quad i = 2, \ldots , K, \quad j=i-1, \\&0, \quad \text {otherwise}, \end{aligned} \right. \end{aligned}$$

and

16 $$\begin{aligned} b_k = \left\{ \begin{aligned}&\frac{\rho _k \gamma _k}{\beta _k} + \frac{\sqrt{{\mathscr {E}}_k}}{c_k} \nu _0, \quad k =1, \\[6pt]&\frac{\rho _k \gamma _k}{\beta _k}, \quad k = 2, \cdots , K. \end{aligned} \right. \end{aligned}$$

The optimization algorithm used in Fit-Seq is L-BFGS-B (Zhu et al. 1997), which is a limited-memory quasi-Newton algorithm for bound-constrained optimization problems. The optimization algorithm used in Fit-Seq2.0 is differential evolution (Storn and Price 1997), which is a population-based metaheuristic search algorithm that is gradient-independent and thus does not require the optimization problem to be differentiable, as is required by quasi-newton methods.

In addition, both Fit-Seq and Fit-Seq2.0 give an estimated lineage trajectory $\{{\hat{r}}_k\}$ for each lineage. In Fit-Seq, the estimated read number of a lineage at $t_k$ is calculated by ${\hat{r}}_k= r_{k-1} {\mathscr {E}}_k R_k / R_{k-1}$ for $k=1, \ldots , K$ and ${\hat{r}}_0= r_0$, with *s* in term ${\mathscr {E}}_k$ (Equation (3)) being the value that optimized. In Fit-Seq2.0, ${\hat{r}}_k$ is calculated by ${\hat{r}}_k= n_k R_k / N_k$ for $k=0, \ldots , K$, with $n_0$ being the value that optimized and $n_k$ for $k=1, \ldots , K$ from Equation (13) given solutions of $\nu _k$.

### Simulation

To evaluate the performance of Fit-Seq2.0 and Fit-Seq, we use a simulated dataset to compare the ground truth in the simulation with the inferred results. Our numerical simulations consider the entire process of a pooled growth experiment of a barcoded cell population using serial batch cultures, which includes five potential sources of noise: cell growth, sampling during cell transfers, genomic DNA extraction, PCR, and sequencing. Specifically, starting from *L* barcodes, with the initial cell number of each barcode following the distribution $f(n_0)$, and the fitness of each barcode following the distribution *f*(*s*), the population grown for *T* generations, with a cell transfer of every $g$ generations of growth. Let $n_i(t)$ be the cell number of lineage *i* at generation *t*, and $s_i$ be the fitness of lineage *i*. For each batch culture cycle, the growth noise is simulated by updating the number of descendants of a single cell according to

17 $$\begin{aligned} n_i(t+1) = {\text {Pois}}\left( \frac{2n_i(t) e^{s_i}}{\sum _i n_i(t) e^{s_i}}\right) . \end{aligned}$$

Here ${\text {Pois}}(\lambda )$ represents a Poisson distribution with parameter $\lambda$. After $g$ generations, the cells which get transferred to the next batch are sampled with

18 $$\begin{aligned} n_i(g) = {\text {Pois}}\left( \frac{n_i(g)}{2^g}\right) . \end{aligned}$$

For each cell transfer time point, 500*L* cells are sampled from the saturated population to simulate the process of genomic DNA extraction and go through 25 rounds of stochastic doubling to simulate PCR with 25 cycles. Then an extra sampling of the size *rL* after PCR is performed to simulate the noise introduced by sequencing, with *r* being the average sequencing read number per lineage per time point. Each step is modeled by a layer of Poisson noise (including for each cycle of PCR). The entire process generates a lineage trajectory over time for each barcode.

Here, $L=10000$, $T=20$, $g=4$, and $r=20, 50, 100$. The distribution of the initial cell number follows the Gamma distribution $f(n0) \sim {\text {Gamma}}(\alpha , \beta )$ with parameters $\alpha =20$ and $\beta =0.2$. Three distributions of fitness are used in the simulations, which are a normal distribution $f(s) \sim {\text {N}}(\mu , \sigma )$ (with mean $\mu =0$ and standard deviation $\sigma =0.15$), a left-skewed normal distribution (with a location parameter of 0, a scale parameter of 0.225, and a skewness parameter of $-3$), and a right-skewed normal distribution (with a location parameter of 0, a scale parameter of 0.225, and a skewness parameter of 3). All fitnesses are normalized and truncated with $-1\le s\le 1$.

## Results

**Fig. 1:**
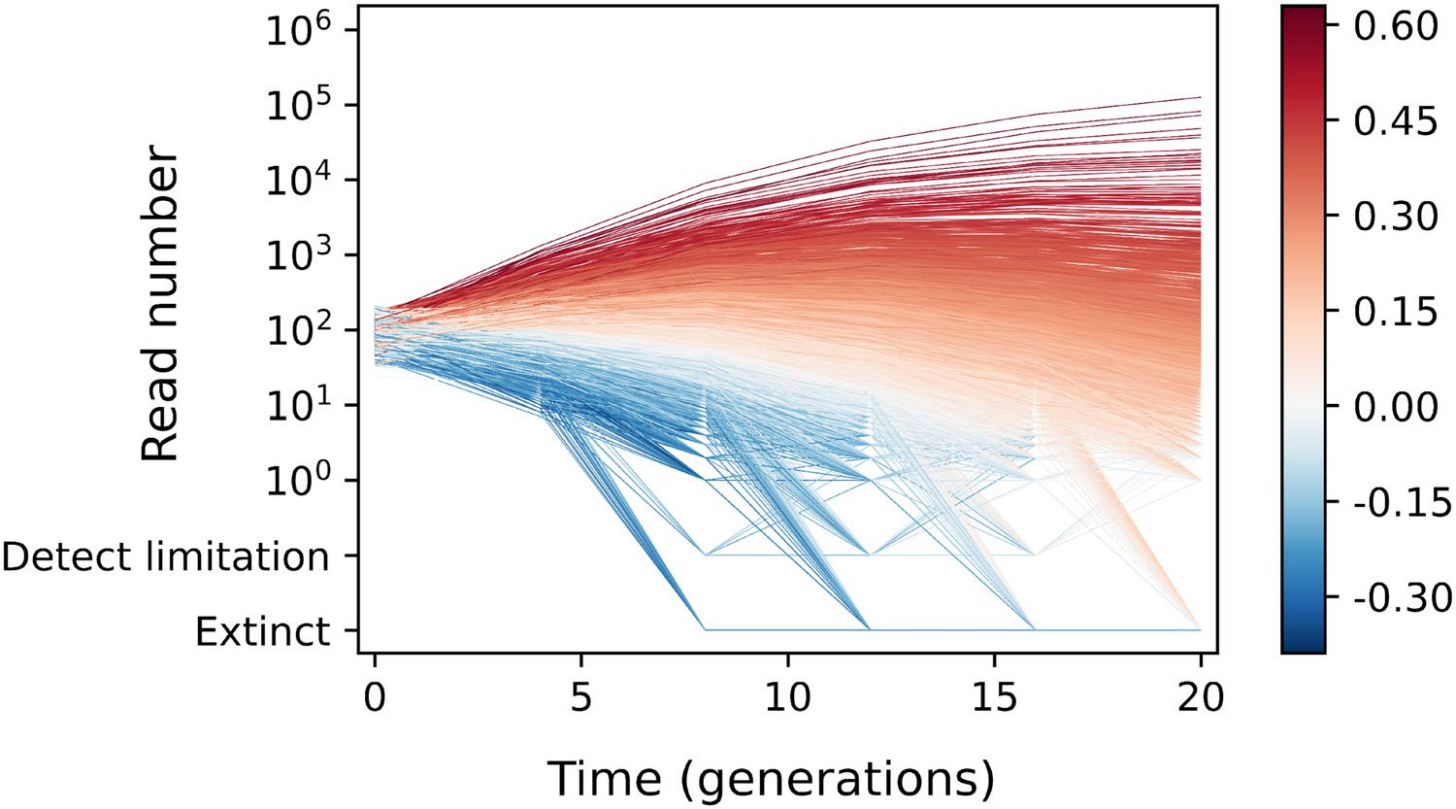
Trajectories of lineages. Lineage trajectories from simulation (corresponds to 3rd row and 3rd column in Fig. 3, Section Simulation). Lineages are colored by their fitnesses (red for fitness $s>0$, and blue for fitness $s<0$)

We simulated fitness re-measurement assays of a barcoded yeast library where the fitness of each lineage is known. These simulations include all sources of experimental noise and the resulting lineage trajectories resemble those generated experimentally. The simulated trajectories of lineages with slightly beneficial variants ($s = 0.0 - 0.15$) in Fig. 1 highlight the major problem with fold enrichment methods, that is, these variants can either enriched or depleted depending on the length of the re-measurement period. These trajectories, containing modestly beneficial variants, begin by increasing in frequency (Fig. 1). However, by later time points, the population mean fitness has increased so that they begin to decrease in frequency, in some cases below their initial frequency. At these later time-points, the fold enrichment methods will erroneously count the modestly beneficial variants as deleterious because they have decreased in frequency.

**Fig. 2:**
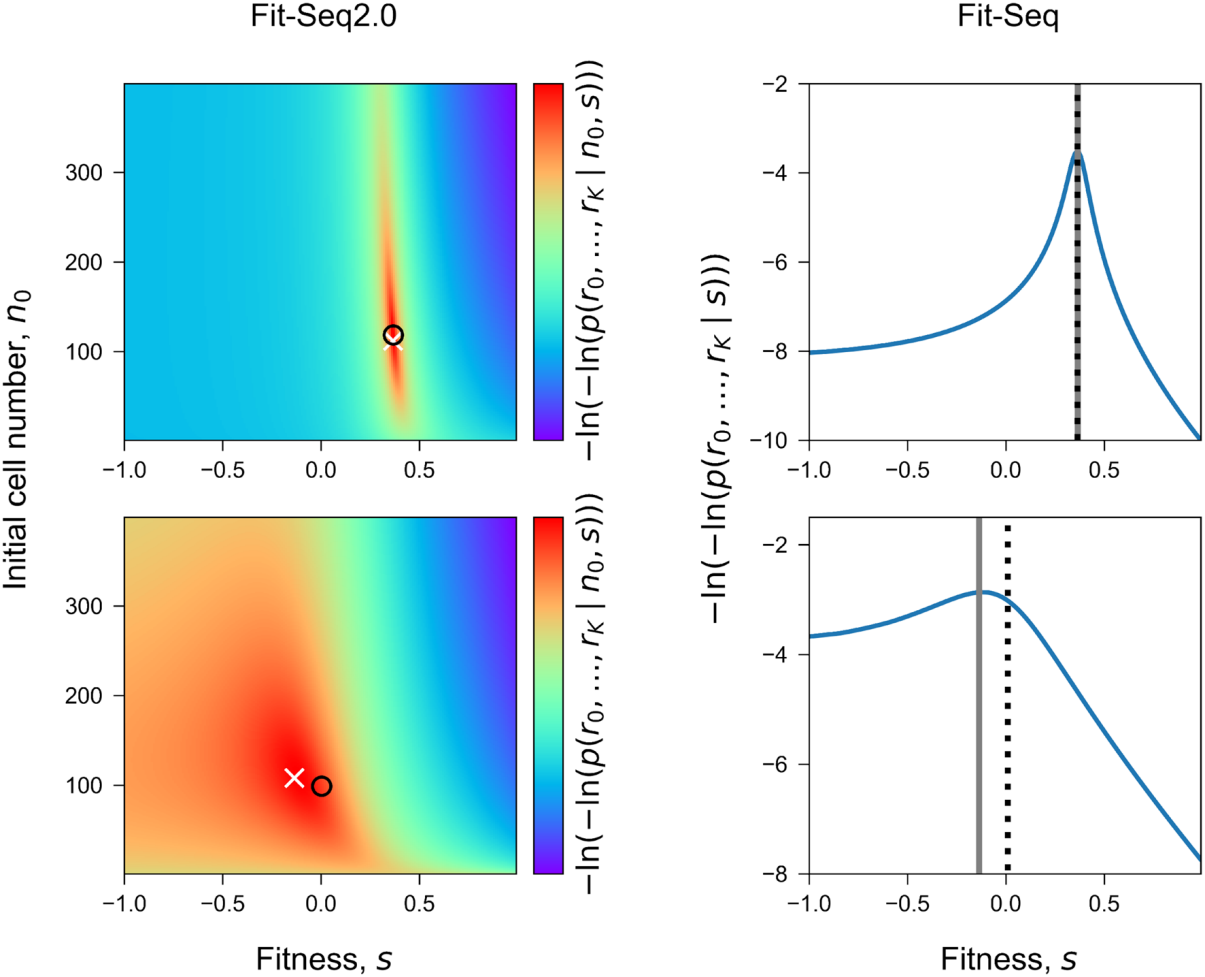
Optimization for two lineages. Optimization results (the last iteration) of the likelihood function in Fit-Seq2.0 (left, Equation (11) and in Fit-Seq (right, Equation (1)) is shown for a lineage with fitness $s>0$ (top) and a lineage with fitness $s<0$ (bottom). Fit-Seq2.0 estimates both the fitness and initial cell number, which is shown by the heatmap of the likelihood function, with true value of fitness and initial cell number marked by $\times$ and optimized result marked by $\circ$. Fit-Seq only estimates the fitness, which is shown by the curve of the likelihood function, with true value of fitness marked by black vertical dashed line and optimized result marked by gray vertical dashed line

Although both Fit-Seq and Fit-Seq2.0 are based on a likelihood maximization method, Fit-Seq2.0 defines a more accurate likelihood function, which models experimental noise more precisely. The likelihood function in Fit-Seq is a single-variable function of the fitness *s*, while the likelihood function in Fit-Seq2.0 is a two-variable function of the fitness *s* and the initial cell number $n_0$. In addition, Fit-Seq2.0 also utilizes an improved optimization algorithm. Together these improve the quality of the fitness estimates. The likelihood functions used in Fit-Seq2.0 and Fit-Seq for a beneficial lineage ($s>0$) and deleterious lineage ($s<0$) are shown in Fig. 2, together with the optimization results. The likelihood function in Fit-Seq2.0 is presented as a heatmap as it has two variables.

**Fig. 3:**
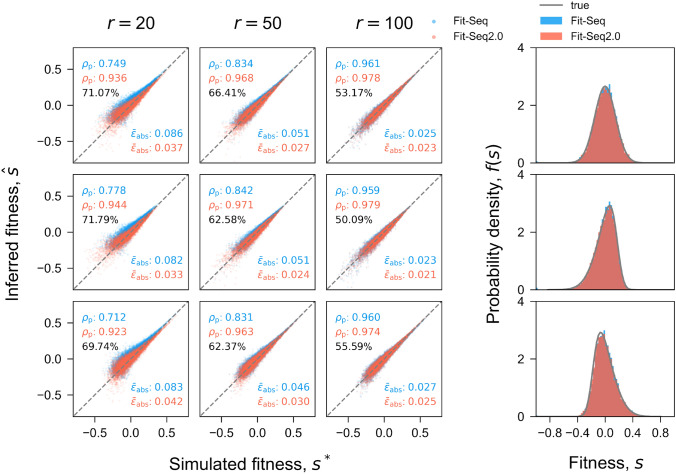
Inference accuracy of the fitness. Comparison of the true fitness in simulation and the fitness inferred by Fit-Seq2.0 (red) and Fit-Seq (blue) for different sequencing read depths (columns) and distributions of fitness (rows). Each panel in the $3 \times 3$ array corresponds to one simulation (Section Simulation). Each point corresponds to a lineage in the simulation. $\rho _{\mathrm{{p}}}$ is the Pearson correlation coefficient. $\epsilon _{\mathrm{{abs}}}$ is the average absolute error, which is defined as $\mid s^* - {\hat{s}}\mid$ for each lineage. The 4th column in shows comparison between the true distribution of the fitness *f*(*s*) in simulation (gray) and the inferred (blue for Fit-Seq and red for Fit-Seq2.0). Percentage is the fraction of lineages with more accurate estimation for the fitness using Fit-Seq2.0. Estimates generated with Fit-Seq2.0 have a higher Pearson correlation coefficient and lower absolute error when compared with Fit-Seq. The distribution of fitness effects estimated by Fit-Seq2.0 also closely matches the true distribution in the simulation

Both Fit-Seq and Fit-Seq2.0 were tested on the simulated data, with both estimates being compared with the true values from the simulation (Fig. 3). The comparison shows that Fit-Seq2.0 has better Pearson correlation coefficients and lower absolute error. These improvements appear to be consistent across a range of initial fitness distributions. It is known that the initial distribution of fitness can impact fitness estimates (Li et al. 2018), because the initial distribution determines how quickly the population mean fitness will increase. Therefore, it is important that any fitness inference algorithm can produce good estimates across a range of initial fitness distributions. Here we tested a normal distribution, a left-skewed normal distribution, and a right-skewed normal distribution. Several empirical studies have found distributions of fitness that follow normal distributions or log-normal distributions which are similar to our left-skewed normal distribution (Sanjuán et al. 2004; Peris et al. 2010; McDonald et al. 2011. In all cases, Fit-Seq2.0 produced better fitness estimates and the distribution of estimated fitness values better matched the true distribution (Fig. 3). The sequencing depth can also impact the fitness estimates. Therefore, we also compared the estimation accuracy of Fit-Seq2.0 and Fit-Seq using simulations with various sequencing read depths, i.e., high ($r=100$), medium ($r=50$), and low ($r=20$). Fit-Seq2.0 resulted in better estimates at all sequencing read depths, and the improvements were the greatest for low depths of sequencing. This means that, by using Fit-Seq2.0, experimenters can now sequence less to produce similar fitness estimates. To further quantify the improvements in Fit-Seq2.0, we also compared the percent of lineages whose fitness estimated is improved using Fit-Seq2.0 instead of Fit-Seq (Fig. 3). Fit-Seq2.0 performs better than Fit-Seq particularly at lower read depths.

**Fig. 4:**
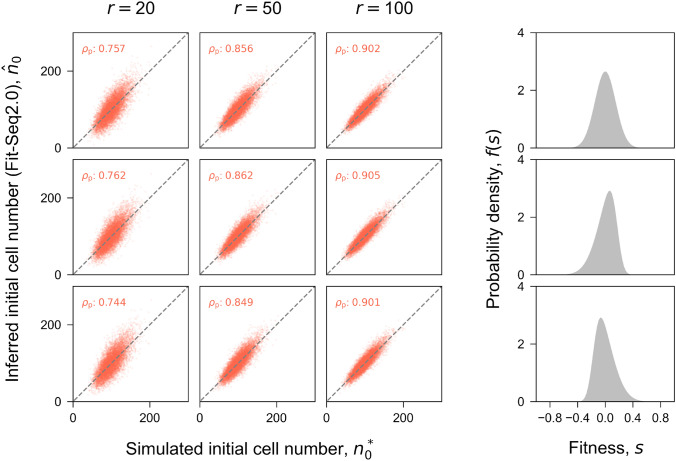
Inference accuracy of the initial cell number. Comparison of the true initial cell number in simulation and the fitness inferred by Fit-Seq2.0 (no estimation for initial cell number in Fit-Seq) for different sequencing read depths (columns) and distributions of fitness (rows). Each panel in the $3 \times 3$ array corresponds to one simulation (Section Simulation). Each point corresponds to a lineage in the simulation. $\rho _{\mathrm{{p}}}$ is the Pearson correlation coefficient. $\epsilon _{\mathrm{{abs}}}$ is the average absolute error, which is defined as $\mid n_0^* - {\hat{n}}_0\mid$ for each lineage. The 4th column in shows the true distribution of the fitness *f*(*s*) in simulation. Estimates generated with Fit-Seq2.0 have a high Pearson correlation coefficient across all conditions that we considered

**Fig. 5:**
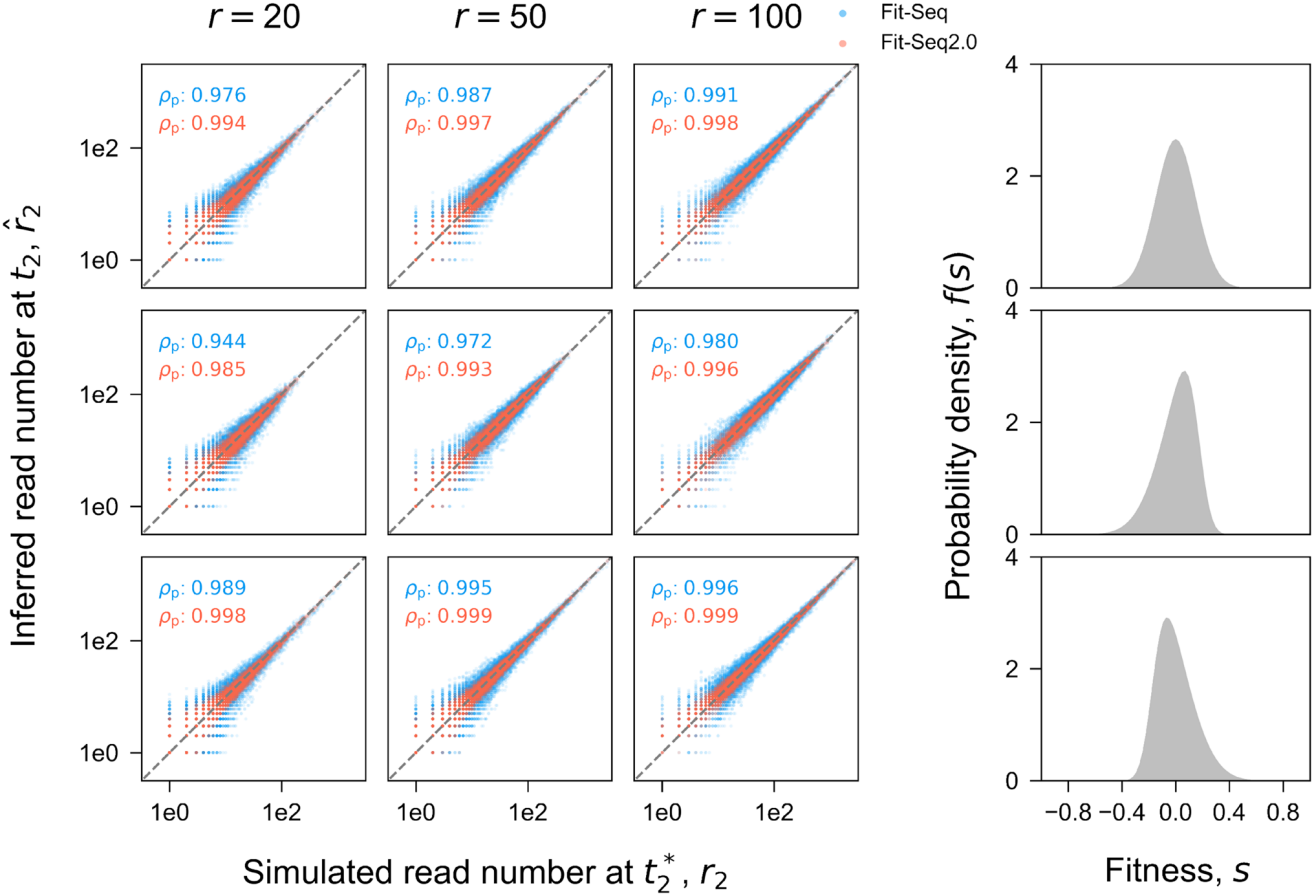
Inference accuracy of the read number. Comparison of the true read number at $t_2=8$ in simulation and the read number inferred by Fit-Seq2.0 (red) and Fit-Seq (blue) for different sequencing read depths (columns) and distributions of fitness (rows). Each panel in the $3 \times 3$ array corresponds to one simulation (Section Simulation). Each point corresponds to a lineage in the simulation. $\rho _{\mathrm{{p}}}$ is the Pearson correlation coefficient. $\epsilon _{\mathrm{{abs}}}$ is the average absolute error, which is defined as $\mid r_2^* - {\hat{r}}_2\mid$ for each lineage. The 4th column in shows the true distribution of the fitness *f*(*s*) in simulation. Estimates generated with Fit-Seq2.0 have a higher Pearson correlation coefficient and lower absolute error when compared with Fit-Seq across all conditions that we considered

Unlike Fit-Seq which only estimates the fitness, Fit-Seq2.0 infers the fitness and the initial cell number simultaneously. The correlation between initial cell number inferred by Fit-Seq2.0 and the true value in the simulation is shown for different distributions of fitness and different sequencing read depths (Fig. 4). The correlation is consistent across different distributions of fitness, while increasing the sequencing read depth improves the inferred initial cell number. Although initial cell number is estimated only in FitSeq2.0, the read number at each time point is estimated in both FitSeq and FitSeq2.0. We show that FitSeq2.0 is better able to estimate the read number at each time point (Fig. 5). This is accomplished by the improved likelihood function and optimization process.

**Fig. 6:**
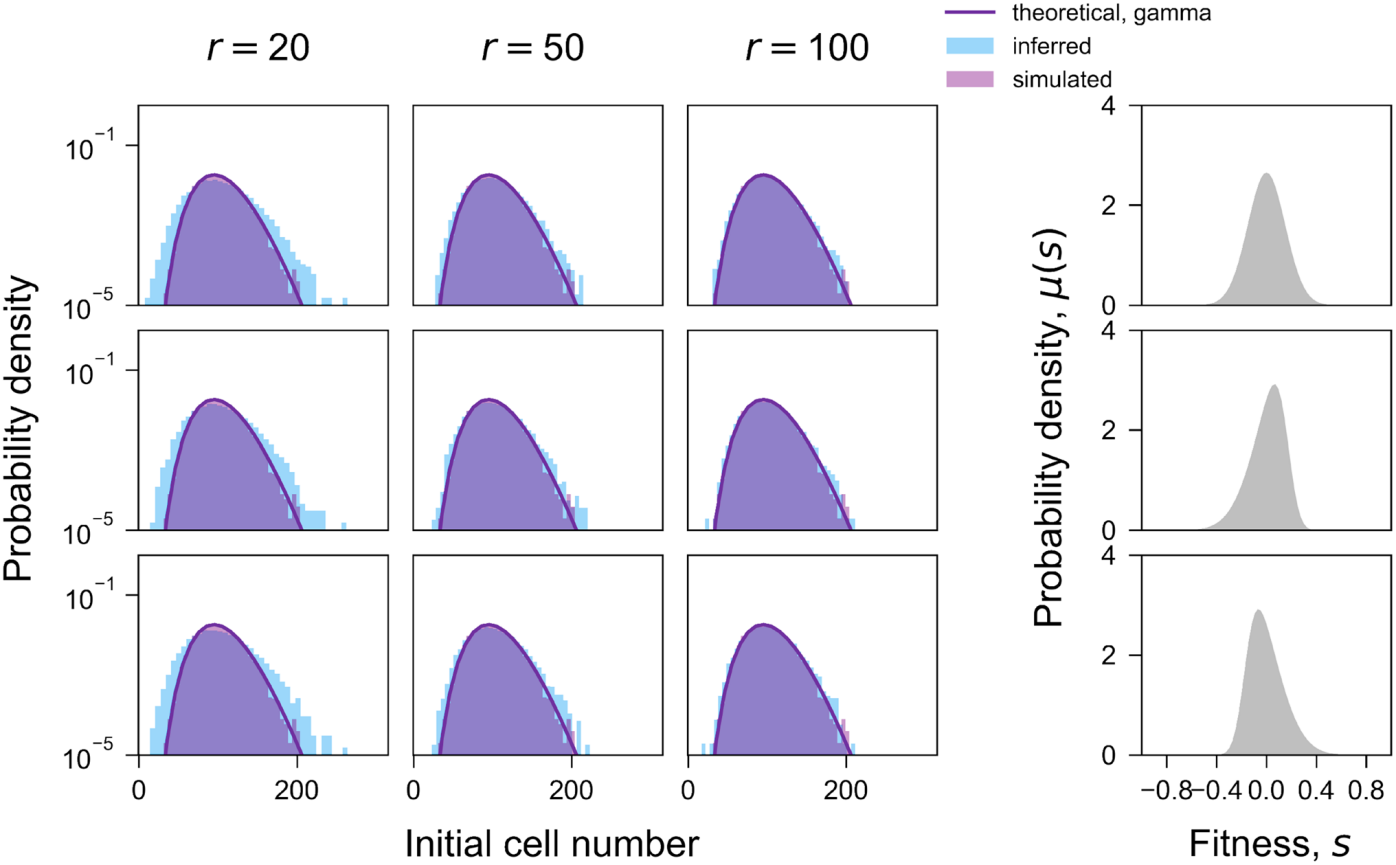
Distribution of initial cell number per lineage. Comparison of the theoretical (purple line), simulated (purple histogram), and inferred (blue histogram) distribution of initial cell number per lineage for different sequencing read depths (columns) and distributions of fitness (rows). Previously simulations have been done using a uniform initial distribution so that every lineage starts with exactly 100 cells

We have also updated some details of the simulations. For simulations in our previous work, the barcoded population started from a population where each barcoded lineage began at the same size (Li et al. 2018). In this work, we used a new approach in the simulations, whereby, the population started with a variable number of cells in each barcoded lineage, which follows a gamma distribution (Fig. 6). This reflects the reality that the initial number of cells for different lineages is usually not the same and therefore better captures how well the algorithm performs on real data. Our updated simulation approach therefore can provide a more robust test dataset for comparison of Fit-Seq and Fit-Seq2.0. The simulated and inferred initial cell number are shown for a range of sequencing read depths and fitness distributions (Fig. 6). We again note that using different fitness distributions makes little difference on the inferences; by contrast, increasing the sequencing read depth improves the inferences.

The compute time of Fit-Seq2.0 and Fit-Seq is approximately on the same order without parallelization. However, the option of parallelization in Fit-Seq2.0 reduces the compute time, which has a negative linear correlation with the number of CPU cores. The compute time of Fit-Seq2.0 depends on both the number of lineages and the number of iterations. A greater number of iterations might be needed when the mean fitness increase very quickly. Here, the per iteration for a simulation of 10000 lineages takes about 2 min when using parallelization (MacBook Pro with Apple M1 chip and 8 G Memory).

We have made our code available at https://github.com/FangfeiLi05/Fit-Seq2.0.

## Discussion

Fit-Seq2.0 is implemented in Python (instead of MATLAB as in Fit-Seq) making it accessible to a wider audience. Both the optimization algorithm and the modeling of experimental noise are improved here, leading to consistent improvements in fitness estimates across a range of fitness distributions and sequencing read depths.

The ability of researchers to accurately and precisely measure fitness is critical in many biological disciplines. For evolutionary biologists, fitness is the phenotype of interest and the ability to measure fitness in high throughput allows the evolutionary process to be understood a way that was not previously possible (Levy et al. 2015; Li et al. 2019). Bulk growth assays are also an increasingly common way for researchers to phenotype large pools of variants (Schubert et al. 2021; Ipsen et al. 2022). However, these data are usually analyzed using a fold enrichment approach, meaning results are difficult to compare across experiments. Instead of fold enrichment, which is dependent on various aspects of the experimental design, such as the time points used, researchers can estimate an unbiased fitness (relative to the initial mean fitness or a reference strain) by utilizing Fit-Seq2.0. Reference strains can be added to each experiments, to estimate fitness relative to the reference, which allows for comparison of results from different large-scale experiments, so that biological insights can be integrated across multiple experimental approaches.

One limitation of Fit-Seq2.0 is that it is under the assumption that the fitness is constant over time. Fit-Seq2.0 is not designed for situations when fitness is changing over time, e.g., frequency-dependent fitness. Another limitation of Fit-Seq2.0 is that the quality of fitness estimates is dependent on the sequencing read depths, with poor fitness estimates below a sequencing depth of 20. Additionally, Fit-Seq2.0 may perform poorly if the distribution of fitness in the pool is too wide and population mean fitness increases very rapidly. Finally, Fit-Seq2.0 is still unable to estimate the confidence intervals for each fitness estimate; however, this is something we aim to incorporate into further updates of Fit-Seq2.0.

The optimization algorithm used in Fit-Seq is L-BFGS-B, which is a gradient-dependent method. This allows us to calculate an estimation error based on the optimization; however, this error is only partially informative of the error associated with the fitness estimates generated. In Fit-Seq2.0, we use a differential evolution optimization algorithm, which is gradient-independent and therefore the estimation of error is not meaningful in this case.
