# Correction: Fit-Seq2.0: An Improved Software for High-Throughput Fitness Measurements Using Pooled Competition Assays

**DOI:** 10.1007/s00239-025-10232-0

**Published:** 2025-02-08

**Authors:** Fangfei Li, Jason Tarkington, Gavin Sherlock

**Affiliations:** Department of Genetics, Stanford University, Stanford, USA

**Correction to: Journal of Molecular Evolution (2023) 91:334–344** 10.1007/s00239-023-10098-0

In this article the legend for Fig. 2 and Fig. 3 was inadvertently swapped.

The original article has been corrected.

